# Mechanical and thermal performances of multistage flexible thermal control device: A case study in cylindrical heat pipe

**DOI:** 10.1016/j.heliyon.2024.e32169

**Published:** 2024-05-31

**Authors:** Min Liu, Xiaoping Fan, Junguang Liu, Ping Li, Yongfeng Zheng, Zhipeng Chen, Jiale Huang

**Affiliations:** a Affiliation: School of Automation, Central South University, Changsha, 410083, China; b Affiliation: School of Mechanical and Electrical Engineering, Guangzhou University, Guangzhou, 510006, China

**Keywords:** Flexible heat pipe, Bionic design, Thermal performance, Parametric study

## Abstract

Multistage flexible heat pipe has been proved to offer advantage of large flexibility as well as low thermal resistance. However, the effects of structural parameters on the comprehensive performances of such multistage thermal control device are still unclear, particularly regarding their mechanical properties. In this paper, effect of structural parameters on the mechanical and thermal performances of bionic multistage heat pipe is investigated. Results show that the stiffness of polymer tubes primarily determines the flexibility of multistage flexible heat pipe. The heat pipe with 4 metal tubes in the adiabatic section can achieve relative large flexibility and maximum bending angle as well as the short start-up time. The bending rigidity of multistage flexible heat pipe increases from 97624.4 N mm^2^ to 293152.9 N mm^2^ when its metal ratio raises from 0 % to 80 %. The thermal resistance of multistage flexible heat pipe decreases more than 32.9 % compared to the traditional flexible heat pipe. When the flexible heat pipe remains straight, the heat transfer performance will slightly increase as the shell metal ratio increases. However, its thermal resistance will also have an additional increase when bending. These results can serve as a guide for the design of the multistage flexible thermal control device.

## Highlights

• Flexible heat pipe with multistage design is constructed and experimental tested.
• Effect of structural parameters on the comprehensive performances of bionic multistage heat pipe is investigated.
• The stiffness of polymer materials dominates the flexibility of bionic flexible heat pipe.
• Flexible heat pipe with 4 metal tubes in adiabatic section can achieve better mechanical and thermal performances.
• Thermal resistance of multistage flexible heat pipe decreases more than 32.9 % compared to the traditional one.

## Nomenclature

**Abbreviations**
PU: Polyurethane
PVC: Polyvinyl chloride

**Symbols**
*A*_v_: Cross-sectional area of channel
C: Constant value depending on the Mach number
*D*: Internal radius of heat pipe
*E*_Cu_: Elastic modulus of copper tube
*E*_PU_: Elastic modulus of polyurethane
*E*_xi_: Maximum measurement error of the independent variable *x*_i_
*E*(*y*): Measurement error of single test
*f*_v_: Friction factor of gas
*g*: Gravitational acceleration
*h*_fg_: Latent heat
*h*_m_: Additional pressure drop
*h*_mi_: Additional pressure drop of each hinge
*h*_mf_: Total additional pressure drop of flexible adiabatic section
*L*_a_: Length of flexible adiabatic section
*L*_eff_: Effective length of heat pipe
*n*: Numbers of copper tubes in the adiabatic section
*P*_0_: Reference pressure (1 atm)
*P*_c_: Vapor pressures of condenser
*P*_e_: Vapor pressures of evaporator
*R*_c_: Gas constant
R: Bending radius
*R*_i_: Bending radius of each hinge
*R*_ec_: Thermal resistance of flexible heat pipe
*R*e_v_: Reynolds number
*r*_h,v_: Diameter of gas channel
*T*_0_: Reference temperature (25 °C)
*T*_1_: Temperature of thermocouple at location 1
*T*_2_: Temperature of thermocouple at location 2
*T*_3_: Temperature of thermocouple at location 3
*T*_4_: Temperature of thermocouple at location 4
*T*_c_: Temperatures of condenser
*T*_e_: Temperatures of evaporator
*x*_i_: Independent variable
*y*: Given function of the independent variable *x*_i_

**Greek symbols**
Δ*P*_v_: Pressure drop of vapor
*θ*: Bending angle
*θ*_i_: Bending angle of each hinge
*μ*_v_: Gas viscosity
*v*: Gas velocity
*υ*_Cu_: Poisson's ratio of copper tube
*υ*_PU_: Poisson's ratio of polyurethane
*ρ*_Cu_: Density of copper tube
*ρ*_PU_: Density of polyurethane
*ρ*_v_: Gas density
*σ*_Cu_: Yield strength of copper tube
*σ*_PU_: Yield strength of polyurethane
*ζ*: Local resistance coefficient of the bend tube
*ω*: Metal ratio

## 1 Introduction

In recent years, the local heating problem of the foldable and wearable devices becomes a severe challenge in electron industry [[1], [2], [3], [4]]. Flexible heat pipes offer considerable flexibility, which can tightly hold on the surface of heat source, flexibly install in complex space and freely move with the motion of heat source. Therefore, they have great potential in the application of flexible/foldable electronic devices [5].

Based on the shell structure, in general, the existing flexible heat pipes can be categorized into single articulated and non-articulated types. The flexibility of single articulated flexible heat pipes is achieved through the flexible adiabatic section. Jaipurkar et al. [6] manufacture a metal flexible heat pipe incorporating stainless steel bellows and calculated its rigidity via finite element method. Results show that the heat pipe can be bent in the range from 5° to 20° during operation. Moreover, the metal bellow not only endures the pressure but also adapts to the change in length because of their expansion properties. Yang et al. [7] conduct experimental tests on the thermal performance of flexible heat pipe containing a polyurethane adiabatic section. Result shows that the bending of heat pipe has little effect on its thermal resistance when the bending angle ranges from 30° to 120°. Yang et al. [8] used fluororubber tube as adiabatic section to fabricate the flexible heat pipe. Results indicated that although the bending will interfere the internal steam flow, its thermal resistance still retains relative low value after bending. Kang et al. [9] designed a squid-like soft heat pipe featuring multiple heat transport branches, which demonstrates both exceptional flexibility and remarkable thermal performance. Result indicated that the squid-like soft heat pipe achieved a maximum equivalent thermal conductivity of 6750 W/(m·K). Similar flexible heat pipe also has great application potential in the field of thermal management garments [10]. Huang et al. [11] proposed a flexible branch heat pipe based on the polyurethane tube, and its maximum bending angle can reach around 90°. However, due to only one flexible connecter in the middle, the deformation range of single articulated heat pipe is limited.

Non-articulated flexible heat pipe can bend at any position along the body due to the low bending rigidity of shell structure. Oshman et al. [12] used laminated sheets of low-density polyethylene terephthalate, aluminum and polyethylene layers as the casing to fabricate the flat flexible polymer heat pipe. Experimental results demonstrated that its effective thermal conductivity is roughly 4.6 times greater than that of copper. Iwata et al. [13] manufactured a micro-Oscillating Heat Pipes (OHP) and used dynamic stiffness to evaluate its flexible heat pipe. Results showed that the OHP was able to operate stably, maintaining constant temperatures in both the evaporator and condenser during and after the stiffness test. Hsieh and Yang [14] manufactured a flexible flat heat pipe made of polymer that could bend within an angle range of 15–90° and found that the minimum thermal resistance occurs at the bending angle of 15°, which even lower than that of straight status. Liu et al. [15] fabrication a flexible flat heat pipe by using aluminum compound packing film as shell structures. The flexible heat pipe can be bent from 0° to 180° and it can retain low thermal resistance of 0.525 °C/W after repeated bending, but the thermal resistance will increase significantly to over 2 °C/W when the bending angle reaches 180°. Lee and Byon [16] experimental tested the thermal performance of pure-metal-based submillimeter-thick flexible flat heat pipe. Results showed that the pure-metal-based flexible heat pipe has an excellent effective thermal conductivity of 3000 W/mK and can realize a certain degree of bending.

Moreover, according to the type of shell materials, the flexible heat pipe also can be divided into pure-polymer-based heat pipes [[12], [13], [14], [15]], pure-metal-based heat pipes [6,16] and the metal-polymer composite heat pipes [7,8,11]. The pure-polymer-based flexible heat pipes could achieve greater flexibility but their thermal performance is relative low, whereas, pure-metal-based flexible heat pipes typically exhibit outstanding thermal performance but sacrifice their flexibility. The metal-polymer composite heat pipes (flexible heat pipes with a single polymer articulated joint) offer both high flexibility and low thermal resistance. However, due to the trade-off between the flexibility and thermal conduction of shell materials, the metal-polymer composite heat pipes still face challenges regarding flexibility, maximum heat transfer power, heat transfer efficiency, stability and so on, limiting their application and widespread use. For example, single articulated flexible heat pipe with polymer adiabatic section can only bend at the hinge and it is easy to failure due to repeated bending. Increasing the length of the polymer adiabatic section can improve the flexibility but also improve the overall thermal resistance and reduce the thermal resistance stability due to the increase of polymer ratio and the larger internal volume variation.

**Fig. 1:**
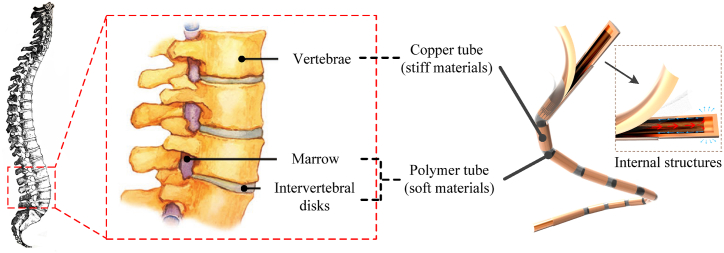
Multistage flexible heat pipe inspired by the human spine [17].

To solve the trade-off between the flexibility and thermal conduction of shell materials, we proposed the multistage flexible heat pipe inspired by the human spine (Fig. 1) in our previous study [17]. Its flexible adiabatic section is composed of copper tubes, polyvinyl chloride (PVC) sealing tapes and heat-shrinkable sleeve. Experimental results showed that compared to the traditional flexible heat pipe, the proposed multistage flexible heat pipe exhibits lower thermal resistance and improved stability after bending. However, the number of sections of metal tubes and the metal ratio of adiabatic section remained fixed, and only the bending angle was considered during experiment for the flexible performance. In fact, bending rigidity and the maximum bending angle are two important mechanical indices for flexible heat pipe when working. Therefore, it is necessary to investigate the effect of structural parameters on the comprehensive performances of multistage heat pipe, especially for the mechanical properties. Here, we use the flexible polyurethane (PU) tube and several copper tubes as the flexible adiabatic section to manufacture the multistage flexible heat pipe. The influence of structural parameters, including the stiffness of polymer and metal tubes, number of copper tubes in adiabatic section, filling ratio of working fluid and metal ratio of adiabatic section, on the mechanical and thermal performance of multistage flexible heat pipe is systematically investigated. This paper can be used to guide the design of bio-inspired multistage flexible thermal control device.

## 2 Materials and methods

### 2.1 Finite element simulation

**Fig. 2:**
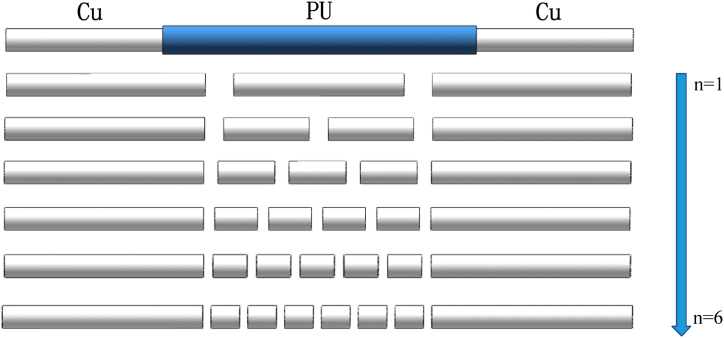
Shell structure of flexible heat pipe with different numbers of copper tubes in the adiabatic section.

The constructed simulated shell of flexible heat pipe is showed in Fig. 2. The external/internal diameters of copper tube are 8/7 mm and the coper tubes of two ends (evaporator and condenser) are 70 mm. The total length of flexible adiabatic section is 80 mm. The external/internal diameters of flexible polyurethane (PU) tube are 10/8 mm and the length is 110 mm. The length of flexible adiabatic section is set as 80 mm in this study. The simulation is carried out in Abaqus/CAE 6.14–1 (Simulia Corp., RI, USA). The mechanical properties of PU and tube are as follows: elastic modulus *E*_PU_ = 100 MPa and *E*_Cu_ = 108 GPa, Poisson's ratio *υ*_PU_ = 0.3 and *υ*_Cu_ = 0.32, yield strength *σ*_PU_ = 39.2 MPa and *σ*_Cu_ = 60 MPa, density *ρ*_PU_ = 2.13 g/cm^3^ and *ρ*_Cu_ = 8.9 g/cm^3^, as shown in Table 1. The PU tubes are bounded to the copper tubes. To calculate the bending rigidity of the flexible heat pipe, the 3D finite element model is restricted at one end, while a vertical displacement of 100 mm is applied to the other end. The C3D8R elements (8-node linear brick, reduced integration, hourglass control) were used in this study and each finite element model contained 209280 elements.

**Table 1:** Mechanical property of PU and copper tubes.

Materials	Elastic modulus (MPa)	Poisson's ratio	Yield strength (MPa)	Density (g/cm^3^)
PU	100	0.3	39.2	2.13
Copper	108 000	0.32	60	8.9

### 2.2 Manufacture process and thermal performance test

**Fig. 3:**
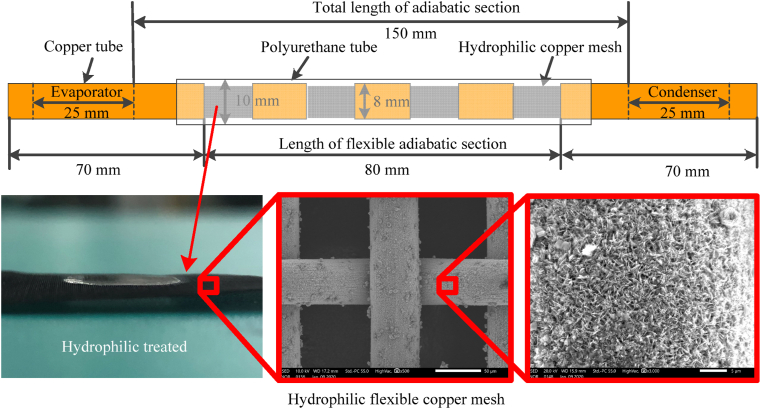
Dimension of multistage flexible heat pipe (n = 3) and the hydrophilic flexible copper mesh.

**Fig. 4:**
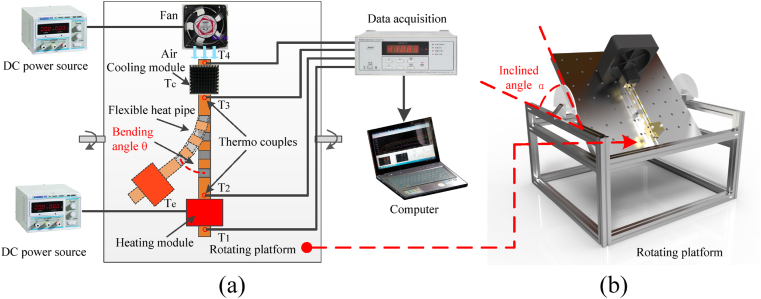
Schematic of (a) experimental setup and (b) rotating platform.

To investigate the thermal performance of flexible heat pipe, the flexible heats with two long copper tubes and a flexible adiabatic section were fabricated. The dimensions of each part of the shell are the same as the finite element model as shown in Fig. 3. The total length of flexible heat pipe is 220 mm and the outer/inner diameters of the copper tube are 8/7 mm. The total length of adiabatic section is 150 mm with the multistage flexible part of 80 mm. The metal ratio is defined as the total length of the metal tubes in flexible adiabatic section divided by the length of multistage flexible part (80 mm). The structural parameters can be easily changed by altering the number and dimension of the copper tubes in the flexible adiabatic section. The heat pipe components are bonded together using a mixture of epoxy resin and hardener in a 1:1 ratio. After curing for one day, the epoxy adhesive forms a strong bond, securely joining each part of the flexible heat pipe. Two layers of hydrophilic copper mesh (300-mesh) are tightly inserted into the inner wall of tube to as served as wick structure (Fig. 3). Detail preparation process of hydrophilic copper mesh can be found in our previous study [17]. The hydrophilic copper mesh can increase the reflux capacity of working fluid and enhance the boiling performance [18]. After exhausting the air, the deionized water (served as working fluid) was injected into the tube. The air is exhausted by the vacuum pump (OERLIKON LEYBOLD vacuum D16C 220V). The vacuum pressure is 20 Pa before injecting working fluid. Ultimately, the flexible heat pipe was provisionally sealed using a heat-shrinkable tube. During the thermal test, the flexible heat pipe and the experimental setup were placed on the rotating platform with the inclined angle *α* from −90° to 90°, as shown in Fig. 4(a and b). Detail process of experimental setup and thermal performance measurement can be found in our previous study [17].

### 2.3 Heat transfer model of multistage flexible heat pipe

Due to the flexible heat pipe can be bent freely, the additional effect of bending on its thermal performance must be considered. In general, bending will lead to the asymmetry fluid flow and heat transfer along the radius direction [[19], [20], [21]]. We neglect this asymmetry and focus on the performance change along the axial direction of the heat pipe in this study. During the calculation of thermal performance of curved heat pipe, the following hypotheses are made according to Yang et al. [8]: (ⅰ) the flexible heat pipe operates steadily; (ⅱ) the vapor or liquid inside the flexible heat pipe is regarded as saturated; (ⅲ) the vapor flow is assumed to be laminar and incompressible; (ⅳ) the liquid in the pipe completely wets the wick structure.

The thermal resistance of flexible heat pipe *R*_ec_ can be calculated as follows.

(1) $$R_{ec}=\frac{T_{e}-T_{c}}{P}$$

where *T*_e_ and *T*_c_ are the temperatures of condenser and evaporator, respectively, and they can be calculated as follows.

(2) $$T_{e}=\frac{T_{1}+T_{2}}{2}$$

(3) $$T_{c}=\frac{T_{3}+T_{4}}{2}$$

where T_1_, T_2_, T_3_ and T_4_ are the temperatures of thermocouples at different locations (Fig. 4a), respectively. By substituting Eq. (2) and Eq. (3) into Eq. (1), the thermal resistance of flexible heat pipe can be obtained.

According to the Clausius-Clapeyron relation, the vapor pressures of condenser (*P*_c_) and evaporator (*P*_e_) can be calculated as follows.

(4) $$P_{C}=P_{0}e^{\left(\frac{h_{fg}}{R_{c}}\frac{T_{C}-T_{0}}{T_{C}T_{0}}\right)}$$

(5) $$P_{e}=P_{c}e^{\left(\frac{h_{fg}}{R_{c}}\frac{T_{e}-T_{c}}{T_{e}T_{c}}\right)}$$

where *T*_0_ and *P*_0_ are the reference temperature (25 °C) and reference pressure (1 atm), respectively. *h*_fg_ is the latent heat, *R*_c_ is the gas constant. The pressure drop of vapor Δ*P*_v_ when steam transfer from evaporator to condenser can be obtained as follows [22].

(6) $$\Delta P_{v}=\left(\frac{C\left(f_{v}-{\text{Re}}_{v}\right)\mu_{v}}{2{\left(r_{h,v}\right)}^{2}A_{v}\rho_{v}h_{fg}}\right)L_{eff}q$$

where *C* is a constant value depending on the Mach number, *f*_v_ is the friction factor of gas, *R*e_v_ is the Reynolds number, *μ*_v_ is the gas viscosity, *r*_h,v_ is the diameter of gas channel, *A*_v_ is the cross-sectional area of channel, *ρ*_v_ is the gas density, *L*_eff_ is the effective length of heat pipe.

The additional pressure drop *h*_m_ due to the bended section can be estimated by the following equations [8,22,23].

(7) $$h_{m}=\varsigma \frac{v^{2}}{2g}$$

(8) $$\varsigma =\left[0.131+0.159{\left(\frac{D}{R}\right)}^{3.5}\right]\frac{\theta }{90{}^{\circ}}$$

where *v* is the gas velocity and *g* is the gravitational acceleration. *D* is the internal radius of heat pipe, *θ* is the bending angle, *R* is the bending radius, *ζ* is the local resistance coefficient of the bend tube. When the vapor arrived to the condenser and changed into liquid, the vapor pressure of condenser *Pc* can be calculated as follows.

(9) $$P_{c}=P_{e}-\Delta P_{v}-h_{m}$$

Combining Eqs. (4), (5), (6), (7), (8), (9), the following equation can be obtained [8].

(10) $$P_{0}e^{\left(\frac{h_{fg}}{R_{c}}\frac{T_{C}-T_{0}}{T_{C}T_{0}}\right)}=P_{C}e^{\left(\frac{h_{fg}}{R_{c}}\frac{T_{e}-T_{c}}{T_{e}T_{c}}\right)}-\left(\frac{C\left(f_{v}-{\text{Re}}_{v}\right)\mu_{v}}{2{\left(r_{h,v}\right)}^{2}A_{v}\rho_{v}h_{fg}}\right)L_{eff}q-\left[0.131+0.159{\left(\frac{D}{R}\right)}^{3.5}\right]\frac{\theta }{90{}^{\circ}}\frac{\nu^{2}}{2g}$$

Eq. (10) shows that the additional pressure drop induced by the bending will increase the temperature of evaporator. Therefore, the temperature difference between the evaporator and condenser will become larger, directly increasing the thermal resistance of heat pipe, as shown in Eq. (1).

For the multistage flexible heat pipe, we assume that the length of flexible adiabatic section is *L*_a_, the metal ratio is *ω* (the length ratio of metal tube to the length of flexible adiabatic section), the number of copper tubes in the adiabatic section is *n*, the bending angle *θ* and bending radius *R*_i_ of each hinge are equal. Therefore, the relation between the total length of plastic tube (*L*_a_) and bending radius of each hinge (*R*_i_) can be obtained as follows.

(11) $$\left(1-\omega \right)L_{a}=n\frac{\theta_{i}\pi }{180{}^{\circ}}R_{i}$$

(12) $$\theta_{i}=\frac{\theta }{n}$$

where *θ*_i_ is the bending angle of each hinge. Combining Eqs. (11), (12), the bending radius *R*_i_ can be calculated as follows.

(13) $$R_{i}=\frac{180{}^{\circ}}{n\theta_{i}\pi }\left(1-\omega \right)L_{a}=\frac{180{}^{\circ}}{\theta \pi }\left(1-\omega \right)L_{a}$$

Therefore, when vapor pass through the single hinge, the additional pressure drop *h*_mi_ can be calculated as follows.

(14) $$h_{\text{mi}}=\frac{v^{2}}{2g}\left\{0.131+0.159{\left[\frac{\theta \pi D}{180{}^{\circ}\left(1-\omega \right)L_{a}}\right]}^{3.5}\right\}\frac{\theta }{90{}^{\circ}n}$$

The total additional pressure drop of flexible adiabatic section *h*_mf_ induced by the bending can be calculated by the following equation.

(15) $$h_{mf}=nh_{mi}=\frac{v^{2}}{2g}\left\{0.131+0.159{\left[\frac{\theta \pi D}{180{}^{\circ}\left(1-\omega \right)L_{a}}\right]}^{3.5}\right\}\frac{\theta }{90{}^{\circ}}$$

Eqs. (14), (15) show that as the number of metal segment increases, the additional pressure drop of single hinge (*h*_mi_) will increase, while the total additional pressure drop keeps constant, indicating that the increased number of hinges will not increase the total thermal resistance of flexible heat pipe. The equations also show that with the increase of metal ratio *ω* and total bending angle *θ*, the pressure drops of single hinge and total flexible adiabatic section are simultaneously increased, leading to the increase of thermal resistance when heat pipe bending. Our previous experiments also prove the increased thermal resistance when heat pipe bending. Results show that the thermal resistance increases around 26.1 % (change from 0.643 °C/W to 0.811 °C/W) when the bending angle increases from 0° to 180° [17]. The metal ratio *ω* will increase the local resistance coefficient of the bend tube *ζ* to increase the total thermal resistance of flexible heat pipe, while the total bending angle will directly increase the additional pressure drop *h*_m_.

### 2.4 Uncertainty analysis of thermal performances

The measurement error of the heating module comes from the DC power supply, which has the 0.05 % voltage accuracy and 0.2 % current accuracy. The precision of the temperature data acquisition module and the calibrated T type thermocouples are 0.1 °C. The primary sources of uncertainty in the testing data are the heating module and the temperature data acquisition module. The relative measurement errors for single-sample test can be calculated as follows [24].

(16) $$\frac{E\left(y\right)}{y}=\frac{\sqrt{\sum_{i=1}^{n}{\left(\frac{\partial y}{\partial x_{i}}E_{xi}\right)}^{2}}}{y}$$

where *E*(*y*) is the measurement error of single test, *y* and *E*_xi_ are the given function and the maximum measurement error of the independent variable *x*_i_, respectively. By substituting the experimental parameters into Eq. (16), the maximum relative measurement uncertainty of the heating powering and the thermal resistance in this study is 0.21 % and 2.19 %, respectively.

## 3 Results and discussions

### 3.1 Effect of structural parameters on the bending rigidity of heat pipes

**Fig. 5:**
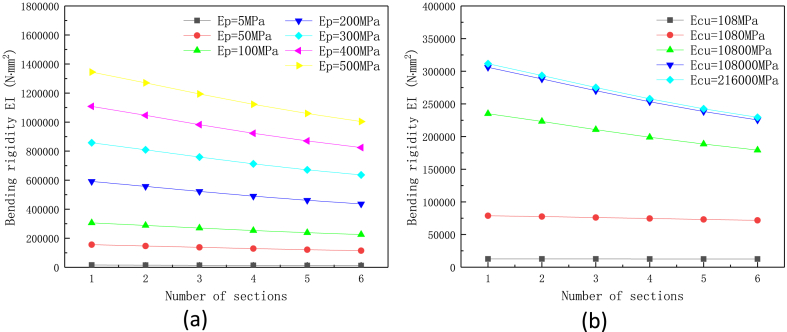
Effect of number of metal tube on the bending rigidity of flexible heat pipe with the changed stiffness of (a) polymer tube and (b) metal tube, the metal ratio ω is set as 75 %.

**Fig. 6:**
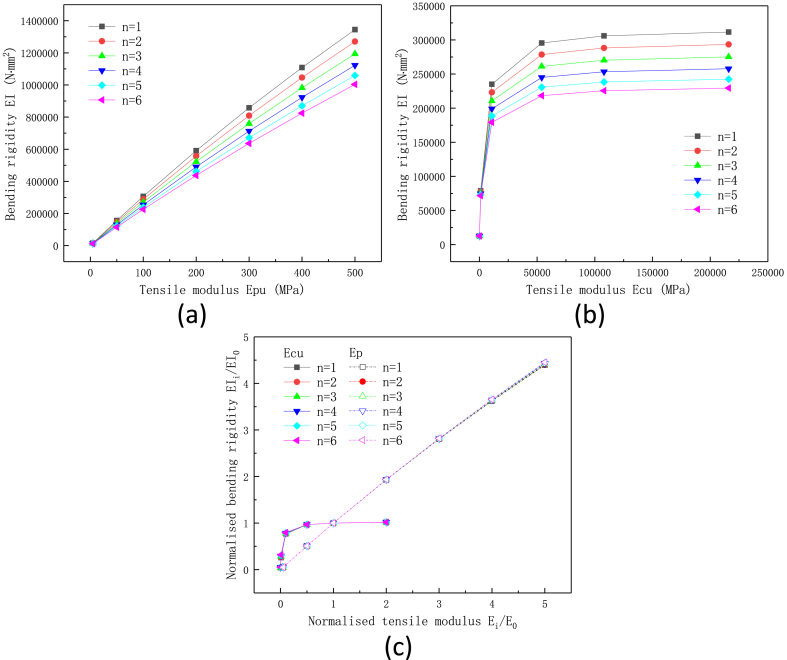
Effect of stiffness of materials on the bending rigidity of flexible heat pipe, the change of (a) stiffness of polymer materials, (b) stiffness of metal materials and (c) normalized stiffness of polymer and metal materials, the metal ratio ω is set as 75 %.

To investigate the effect of numbers of tubes and stiffness of materials on the flexibility of heat pipes, the total length of the metal tube is set as 60 mm (the corresponding metal ratio ω is 75 %). The results are shown in Fig. 5 (a, b) and Fig. 6 (a, b, c). The plot shows that the bending rigidity of flexible heat pipe is significantly decreased as the number of metal tubes increase. When the numbers of metal tubes increase from 1 to 6, the shell bending rigidity of heat pipe decrease by 25.33 %–26.40 % under different stiffness of polymer materials (*E*_PU_ ranges from 5 MPa to 500 MPa), indicating that the effects of number of metal tube and the stiffness of polymer materials are independent to each other. For the metal materials, the influence of stiffness is more complicated. When the tensile modulus of metal tube *E*_Cu_ (rigid tube) is in the range of 10.8 GPa–216 GPa, the bending rigidity of shell decreases by 23.74 %–26.37 % as the number of metal tubes increases from 1 to 6. When the stiffness of rigid tubes is 1.08 GPa (in the range of some polymer materials), the decrease of stiffness is only 9.03 % due to the number increase of metal tubes. When the stiffness of metal tube (108 MPa) is close to the polymer tubes (100 MPa), the number of metal tubes has little effect on the overall bending rigidity of flexible heat pipe (the decrease is only 0.40 %). It can be seen that when the rigid material is metal (generally greater than 45 GPa), the effects of the metal stiffness and the number of tubes on the flexibility of the multistage heat pipe are almost independent of each other. Meanwhile, when the stiffness of the metal material is low and close to the flexible polymer material, the influence of number of metal tubes on the total stiffness of the heat pipe is almost negligible (Fig. 5b). Fig. 6c shows the effect of normalized stiffness of polymer and metal materials on the bending rigidity of flexible heat pipe. The reference tensile moduli of polymer and metal materials are 100 MPa and 108 GPa, respectively. The plot shows that the slope of polymer curve is much larger than that of the metal curve, indicating the higher sensitivity of polymer stiffness on the total bending rigidity than metal stiffness. Therefore, the stiffness of the polymer material dominates the total stiffness of the flexible heat pipe, and it is more effective to alter the stiffness of the flexible material for regulating the total stiffness of flexible heat pipe.

**Fig. 7:**
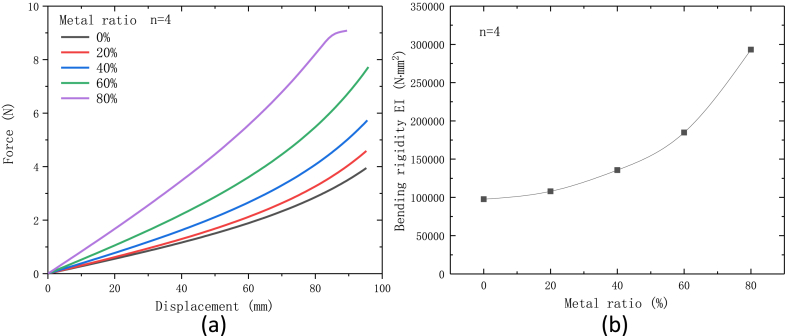
(a) Force-displacement curve and (b) bending rigidity of flexible heat pipe with different metal ratios when n = 4.

Effect of the metal ratio on the mechanical properties of flexible is shown in Fig. 7(a and b). The plot shows an exponential trend of increment. When the metal ratio of adiabatic section is 0, the bending rigidity of flexible heat pipe is around 97624.4 N mm^2^. As the metal ratio increases, the bending rigidity increases rapidly and it reaches around 293152.9 N mm^2^ when the metal ratio is equal to 80 %. Therefore, increasing the metal ratio of adiabatic section will significantly decrease the flexibility of flexible heat pipe.

### 3.2 Effect of the numbers of metal tubes on the maximum bending angle

**Fig. 8:**
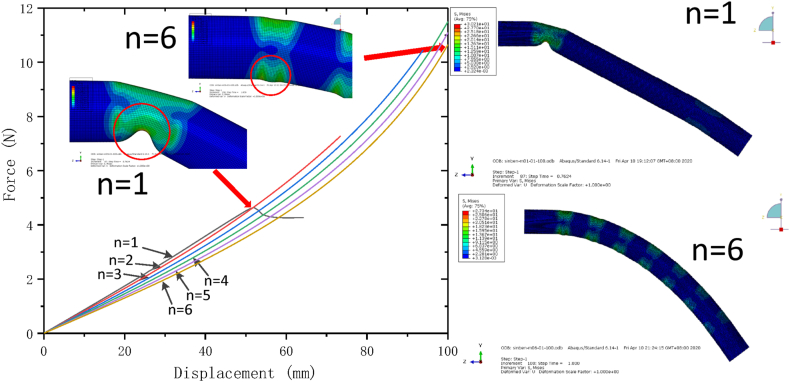
Force-displacement curve of flexible heat pipe with different numbers of metal tubes, the metal ratio ω is set as 75 %.

**Fig. 9:**
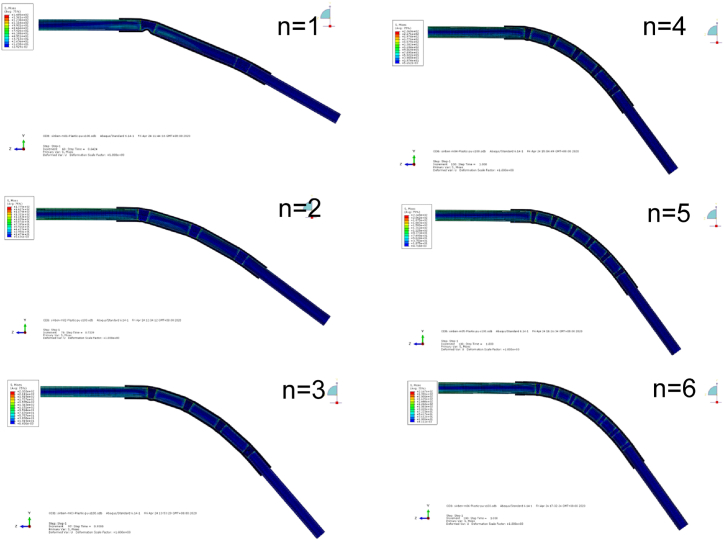
Maximum bending deformation contour plot of flexible heat pipes with different numbers of metal tubes, the metal ratio ω is set as 75 %.

**Fig. 10:**
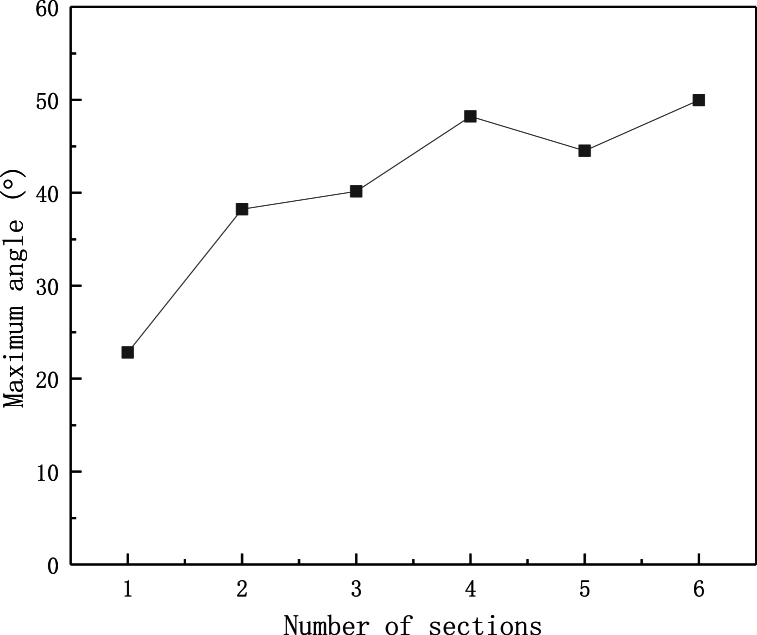
Maximum bending angle of flexible heat pipes with different numbers of metal tubes, the metal ratio ω is set as 75 %.

In addition to the bending rigidity, the maximum bending angle is also an important index for evaluation the mechanical performance of flexible heat pipe. The multistage flexible heat pipes with different numbers of metal tubes are constructed and simulated under the loading condition of concentrated force. Results are shown in Fig. 8, Fig. 9. The plot shows that when the number of copper tube is small, the flexible heat pipe is easy to undergo severe local instability at the first joint. The wrinkle of the flexible heat pipe resulting from the local buckling is relieved as the number of copper tubes increases. Therefore, its maximum bending angle stably increases, as shown in Fig. 9, Fig. 10. The plot also shows that when the number of metal tubes increases from 1 to 4, the maximum bending angle increases significantly (from 22.82° to 48.22°), while the maximum bending angle increases slightly (from 48.22° to 49.97°) and gradually trends to be stable when the number of metal tubes is larger than 4.

**Fig. 11:**
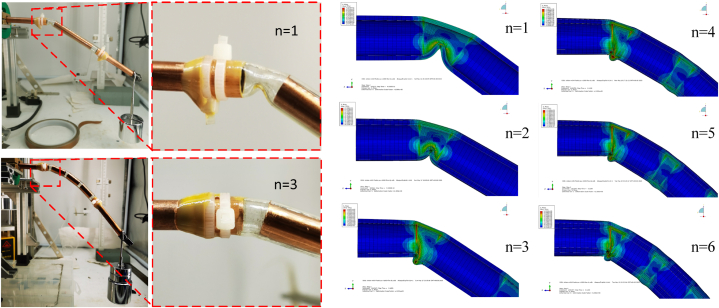
The deformation modes of the flexible heat pipe at the first joint, the metal ratio ω is set as 75 %.

**Fig. 12:**
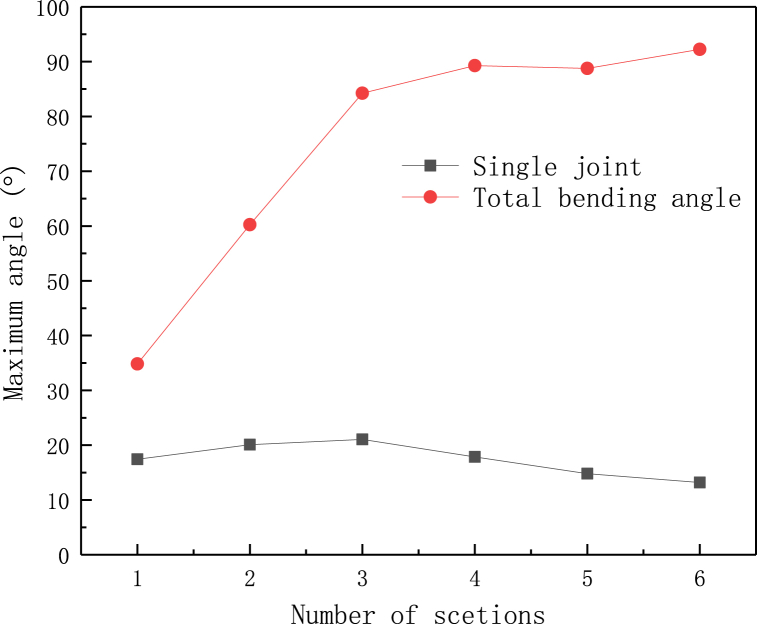
The maximum bending angle of flexible heat pipe at the first joint and the predicting maximum bending angle of the total flexible heat pipe, the metal ratio ω is set as 75 %.

The deformation of the first joint in Fig. 11 shows that the deformation mode of the shell of flexible heat pipe changes as the number of metal tubes varies. When the number of metal tubes *n* is small (1 - 2 sections), the flexible shell is local buckling inward. Because the wick structure of copper mesh is tightly attached to the inner surface of the copper tubes, this failure will press the wick structure, disturbing the distribution of liquid and gas flow fields. As the number of metal tubes increases, the deformation of the first joint alters from inward to outward, and the degree and amplitude of the local buckling also decreases, which has no directly effect on the wick structure, benefitting the stability of internal flow field and improving the stability of thermal resistance of flexible heat pipe. This phenomenon also can be observed by the experiment. The flexible shell is local buckling inward at n = 1 and it changes from inward to outward at n = 3 (Fig. 11). The maximum bending angle of single joint (corresponding to the bending angle of first joint) of flexible heat pipe is shown in Fig. 12. The maximum bending angle of single joint increases initially and then it decreases as the number of metal tubes increases. When the number of metal tubes is 3, the maximum bending angle of single joint reaches the maximum value of 21.06°. The product of maximum bending angle of single joint and the number of joints can be used to predict the maximum bending angle of the total flexible heat pipe, as shown in Fig. 12. The plot shows that when the number of metal tubes is 4 (the corresponding number of joints is 5), the maximum bending angle of total flexible heat pipe is 89.27°, which is only slightly less than that of 6 (92.24°). Therefore, when the number of metal tubes is equal to 4, the flexible heat pipe can achieve an relatively excellent mechanical property.

### 3.3 Effect of filling ratio on the thermal performance of flexible heat pipe

**Fig. 13:**
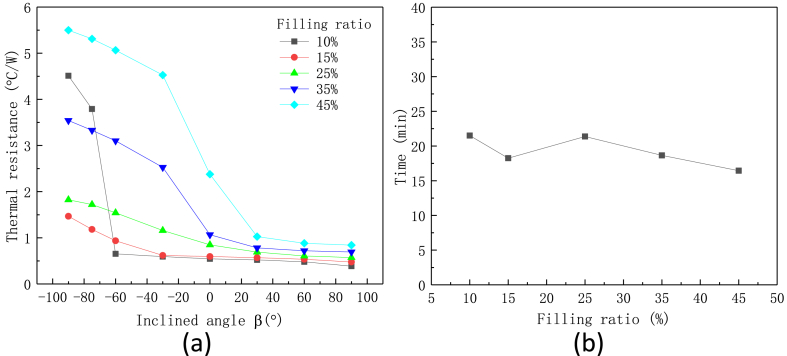
Effect of filling ratio on the thermal performances of multistage flexible heat pipe, (a) thermal resistance and (b) start-up time, the metal ratio ω is set as 75 %.

Effect of filling ratio of working fluid on the thermal performance of flexible heat pipe with 4 metal tubes and 75 % metal ratio at the adiabatic section is shown in Fig. 13(a and b). The heating power is 10 W. We assume the heat pipe has reached a stable state when the temperature fluctuation of the evaporator over the last 10-min interval is less than 2 %. The corresponding time between the initial heating stage and the finial stable stage is defined as start-up time. The filling ratio is defined as the volume ratio of the working fluid to the total internal hole space of the tube. Because the evaporator will dry out when the filling ratio is equal to 5 %, the thermal performance of flexible heat pipe with the filling ratio of 15 %–45 % is tested. The plot shows that the thermal resistance decreases with the decrease of filling ratio. The thermal performance of flexible heat pipe with 10 % filling ratio is comparable to that with 15 % filling ratio, but heat pipe with 10 % filling ratio will be dry-out and even invalid under the large anti-gravity angle, leading to the large thermal resistance. Whereas the thermal resistance of flexible heat pipe with 15 % is stable and keeps a low value under different inclined angle. For the heat pipe with the filling ratio larger than 35 %, its thermal resistance is small only under the gravity-assistance condition (inclined angle >0°). Under the anti-gravity condition, its thermal resistance increases rapidly because plenty of free water exits in the internal space and it is easy to gather in the condenser, dramatically deteriorating the thermal performance. The start-up time of flexible heat pipe with different filling ratios is shown in Fig. 13b. The plot shows that the start-up time has a slow and fluctuating downward trend as the filling ratio increases. Considering the heat transfer performance, the anti-gravity ability and the start-up time, flexible heat pipes with 15 % filling ratio are used in the following discussion.

### 3.4 Effect of number of metal tubes on the thermal performance

**Fig. 14:**
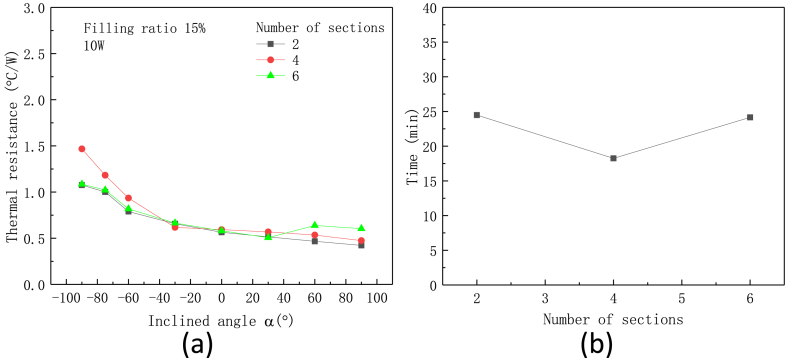
Effect of number of metal tubes on the thermal performance of flexible heat pipe, (a) thermal resistance and (b) start-up time, the metal ratio ω is set as 75 %.

Effect of number of metal tube on the thermal performance of flexible heat pipe with the metal ratio of 75 % under the heating power of 10 W is show in Fig. 14(a and b). Unlike the significant influence on the flexibility and maximum bending angle, the number of metal tube almost has no effect on the thermal resistance of the flexible heat pipe. The thermal resistances of heat pipe with different number of metal tubes are almost the same especially when the heat pipes are positioned horizontally. However, the flexible heat pipe with 4 metal tubes exhibits the quickest start-up time, and its start-up time is 25 % shorter than those with 2 and 6 metal tubes, as shown in Fig. 14b.

### 3.5 Effect of metal ratio on the thermal performance

**Fig. 15:**
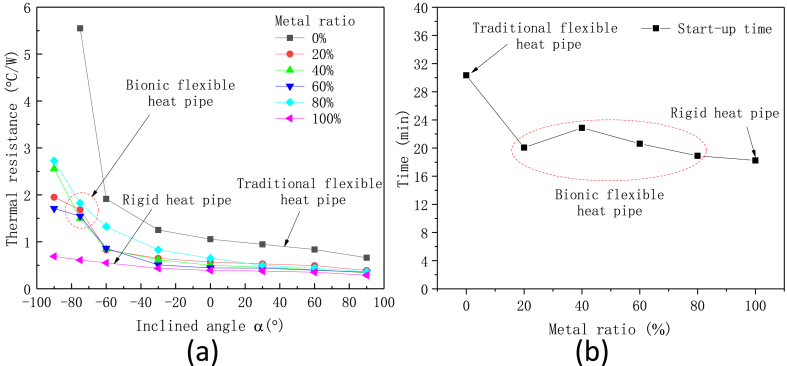
Effect of metal ratio on the thermal performance of multistage flexible heat pipe, (a) thermal resistance and (b) start-up time.

To investigate the effect of metal ratio on the thermal performance of multistage flexible heat pipe, heat pipes with 4 metal tubes were used in the tests. The metal ratio ranges from 20 % to 80 % and the experimental results are shown in Fig. 15(a and b). The plot shows that the values of thermal resistances of bionic flexible heat pipes are located in the range between the rigid heat pipe and the traditional flexible heat pipe, and it is much closed to the rigidity heat pipe, especially under the gravity-assistance condition. The start-up times of flexible heat pipe with different metal ratios are shown in Fig. 15b. The plot shows that the start-up times of bionic flexible heat pipes are only slightly longer than that of rigid heat pipe and much less than that of traditional heat pipe. The start-up time of heat pipe with 80 % metal ratio is reduced to 18.9 min, which is decreased by 37.69 % compared to the traditional heat pipe, and it is comparable to the rigid heat pipe.

**Fig. 16:**
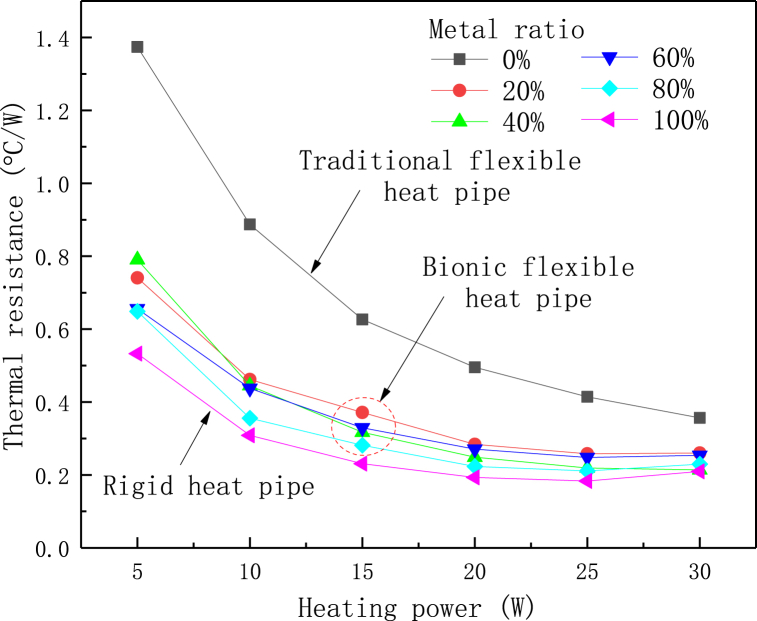
Effect of heating power on the thermal resistance of multistage flexible heat pipe.

In addition to the filling ratio and inclined angle, heating power also has important influence on the thermal performance of heat pipe. The effect of heating power on the thermal performance of flexible heat pipe with different metal ratios in the straight state is shown in Fig. 16. The plot shows that under different heating powers, the thermal resistance of bionic flexible heat pipe decreases significantly and reaches the minimum value at the heating power of 25 W. When the heating powering increases to 30 W, the thermal resistance almost keeps constant or slightly increases. Therefore, the best heat transfer performances of theses flexible heat pipes occur at the heating power of 25 W. Moreover, when the heating power is 5 W, the thermal resistance of multistage flexible heat pipe decreases around 48.4 % compared to the traditional flexible heat pipe, and this decrease is greater than that at the heating power of 30 W (decreases around 32.9 %). The heat transfer path of the heat pipe consists of two parts. The majority of the heat is transferred via the phase change process in the vapor chamber, while the remaining heat is conducted through the shell. When the heating power is low, the boiling of working fluid in the flexible heat pipe is relatively weak. The ratio of shell conduction to the overall heat transfer is relatively large. Thus, the heat transfer performance will slightly increase as the shell metal ratio increases. With the increase of heating power, the phase-changed process achieves absolute dominance in the heat transfer process, and the importance of thermal conduction of the shell continues to reduce. Therefore, the reduction of thermal resistance induced by the increase of metal ratio is relative small at the high heating power. Note that for the bionic flexible heat pipe, increasing the metal ratio can only slightly increase its thermal performance in the straight state. However, its thermal resistance will also have an additional increase when bending, according to Eq. (13). Moreover, the flexibility of bionic flexible heat pipe will significantly decreases as the metal ratio increases (Fig. 7). Therefore, it may not be a good way to improve the heat transfer performance of flexible heat pipes by simply changing the metal ratio in adiabatic section.

## 4 Conclusions

In this paper, the influence of structural parameters, including the stiffness of polymer and metal tubes, number of copper tubes in adiabatic section, metal ratio of adiabatic section and filling ratio of working fluid, on the mechanical and thermal performance of multistage flexible heat pipe is systematically investigated through numerical simulation and experiment. The following conclusions can be made.

(1) The stiffness of polymer materials dominates the flexibility of bionic flexible heat pipe. The bending rigidity of flexible heat pipe increases from 97624.4 N mm^2^ to 293152.9 N mm^2^ when the metal ratio increases from 0 % to 80 %.
(2) The flexibility of shell increases with the increase of number of metal tubes. The heat pipe with more than 4 metal tubes in the adiabatic section can achieve relative large flexibility and maximum bending angle. The number of metal tubes in the adiabatic section has little effect on its thermal resistance, but heat pipe with 4 metal tubes has shorter start-up time compared to those with 2 and 6 metal tubes.
(3) Thermal resistance of multistage flexible heat pipe decreases more than 32.9 % compared to the traditional flexible heat pipe. When the flexible heat pipe keeps the straight state, the heat transfer performance will slightly increase as the shell metal ratio increases. However, its thermal resistance will also have an additional increase when bending according to the theoretical prediction. Therefore, it may not be a good way to improve the heat transfer performance of flexible heat pipes by simply changing the metal ratio in adiabatic section.

## Data availability statement

The data supporting this study's findings are available from the corresponding author upon reasonable request.

## CRediT authorship contribution statement

**Min Liu:** Writing – original draft, Investigation, Data curation. **Xiaoping Fan:** Data curation. **Junguang Liu:** Data curation. **Ping Li:** Data curation. **Yongfeng Zheng:** Data curation. **Zhipeng Chen:** Data curation. **Jiale Huang:** Writing – review & editing, Writing – original draft, Visualization, Validation, Supervision, Methodology, Investigation, Formal analysis, Data curation, Conceptualization.

## Declaration of competing interest

The authors declare the following financial interests/personal relationships which may be considered as potential competing interests:Jiale Huang reports financial support was provided by 10.13039/501100014881Guangzhou University. 52005422 reports a relationship with 10.13039/501100001809National Natural Science Foundation of China that includes: funding grants. None If there are other authors, they declare that they have no known competing financial interests or personal relationships that could have appeared to influence the work reported in this paper.
